# Metagenomic analysis of tomato rhizosphere microbiomes detects *Ralstonia* phage sequences in Kenyan agricultural soils

**DOI:** 10.1371/journal.pone.0362106

**Published:** 2026-11-04

**Authors:** Derleen Bosibori Mogire, Johnstone Neondo, Christabel Muhonja, Consolate Awuor, Daniel Mureithi, Stephen Ogada, Tofick Barasa Wekesa

**Affiliations:** 1 Institute for Biotechnology Research, Jomo Kenyatta University of Agriculture and Technology, Nairobi, Kenya; 2 Biological Sciences, Machakos University, Machakos, Kenya; 3 Health Sciences, Machakos University, Machakos, Kenya; 4 Agricultural Sciences, Machakos University, Machakos, Kenya; NSTRI: Nuclear Science and Technology Research Institute, IRAN, ISLAMIC REPUBLIC OF

## Abstract

Bacterial wilt caused by *Ralstonia solanacearum* species complex is a major constraint in tomato production in Kenya, resulting in yield losses of up to 100% in heavily infested fields. This study used high-throughput shotgun metagenomic sequencing approach to characterize the rhizosphere microbiomes of tomato plants in three of the major production areas in Kenya (Kirinyaga, Kiambu, and Laikipia counties) focusing on detecting *Ralstonia* phage sequences. Soil samples were collected from the rhizosphere of bacterial wilt-infected tomato plants, and microbial DNA was extracted using a modified phenol-chloroform protocol and sequenced using Illumina NovaSeq platform. Taxonomic profiling revealed diverse microbial communities dominated by Actinobacteria and Proteobacteria. Four *Ralstonia* species were confirmed (*R. insidiosa*, *R. pickettii*, *R. mannitolilytica* and *R. wenshanensis*). Sequences similar to the *R. solanacearum* species complex were also detected but were too short for species-level confirmation. Metagenomic analysis detected sequences matching four previously described *Ralstonia* phages (RSL1, RSIBR3, Anchaing, and Cimandef). Functional annotation demonstrated abundant metabolic pathways, energy production systems, and stress response mechanisms, indicating highly adaptive microbial communities in the tomato rhizosphere. This study provides baseline data on rhizosphere microbiome composition in Kenyan tomato production systems and identifies *Ralstonia* phage sequences that could guide future isolation and host-range studies. If future work shows that these phages are active against *Ralstonia*, they could contribute to locally adapted biocontrol approaches that reduce reliance on chemical pesticides.

## 1 Introduction

Tomato (*Solanum lycopersicum*), a member of the *Solanaceae* family, is one of the world’s most economically valuable horticultural crops [1,2]. Its global importance derives from nutritional properties particularly high concentrations of lycopene, beta-carotene, vitamins A, C, and E, which contribute to its health benefits, [3,4]. One of the antioxidants found in tomatoes, lycopene, has been associated with reduced risks of chronic diseases such as cancer and cardiovascular disease [4]. Additionally, tomatoes are also widely consumed in various forms, from fresh to processed items, without any loss of nutritional value [4].

Tomato farming in Kenya plays a vital role in the sustenance of small-scale farmers, creating a source of income in different agroecological areas [5,6]. The areas range from the highland humid areas surrounding Mount Kenya to semi-arid areas in Eastern Province, where farmers use different methods of production to increase yield and marketability [7].

Despite its agricultural significance, tomato production faces devastating constraints from *Ralstonia solanacearum*, the causal agent of bacterial wilt disease [8]. *R. solanacearum* is considered the second most destructive plant pathogenic bacterium, ranking near the top of the list of the world’s most devastating bacterial plant pathogens [9]. This soil-borne pathogen results in catastrophic economic losses of over $1 billion annually worldwide [8,10]. Bacterial wilt has a severe effect on tomato yields in Kenya, resulting in losses of up to 100% in highly infested regions [11]. Embu, Murang’a, Nakuru, Nyahururu, and Kirinyaga report the highest prevalence and incidence, marking them as major hotspots for the disease [12]. The pathogen’s exceptional virulence is attributed to several factors, including its soil persistence, allowing survival for several years without a host [13]. Additionally, *R. solanacearum* also displays vast genetic diversity with five biovars, five races, and four phylotypes [14,15] and a wide host range with more than 250 plant species in 54 botanical families [16,17].

Climate change has intensified this agricultural threat by expanding *R. solanacearum’s* geographical distribution and potentially enhancing its virulence [18]. Rising soil temperatures and altered precipitation patterns create favorable conditions for bacterial proliferation and disease expression in previously unsuitable regions [18]. The management of bacterial wilt in tomatoes presents significant challenges, as no single method has demonstrated complete effectiveness in combating the disease to date [19]. While bactericides such as copper and antibiotics including streptomycin, ampicillin, tetracycline, and penicillin have been employed, their efficacy in reducing *R*. *solanacearum* populations in agricultural settings is limited, and their application poses environmental risks and incurs high costs [19]. Furthermore, documented cases of antimicrobial resistance development in *R. solanacearum* populations underscore the urgent need for alternative control strategies [20,21]. Additionally, this bacterial pathogen affects other members of the *Solanaceae* family, such as peppers, potatoes, and eggplant, thereby making crop rotation an undesirable control strategy [19,22]. Consequently, an integrated pest management strategy that incorporates a variety of control measures such as enhancing host resistance, implementing cultural practices, and utilizing both chemical and biological controls, is recommended for effectively managing bacterial wilt in regions where the pathogen is prevalent [19].

Bacteriophages offer an effective, environmentally friendly alternative to conventional chemical control. Their targeted approach preserves beneficial microorganisms in the soil, while self-replication enables constant presence where infection is present, increasing effectiveness with time [23]. Recent research confirms that phage-based biocontrol strategies effectively reduce bacterial wilt occurrence in crops, with increased phage concentrations in the rhizosphere correlating to enhanced soil suppressiveness against *R. solanacearum*, ultimately advancing disease management capabilities [24].

The rhizosphere, a 1–2 mm soil zone influenced by root exudates functions as a crucial interface where plant-microbe interactions determine disease outcomes [25–27]. This ecological niche contains rich microbial diversity, including potential antagonists to *R. solanacearum* [28]. While traditional culture methods detect only 0.1–1% of soil microbes, metagenomic approaches provide unbiased, comprehensive characterization of microbial communities, revealing their interactions and contributions to plant health [29,30]. This root-soil interface affects plant development, stress responses, and ecosystem functioning [31,32], making metagenomic analysis an important tool to investigate both the taxonomic composition and functional potential of these communities. However, little is known about the composition and functional potential of microbial and viral communities in Kenyan tomato rhizospheres, particularly their role in natural suppression of *Ralstonia* spp. This limits the development of locally adapted biocontrol strategies guided by rhizosphere microbiome composition.

The aim of this study is to characterize the taxonomic and functional profiles of tomato rhizosphere microbiomes in Kenya, using shotgun metagenomic sequencing, with emphasis on detecting sequences related to bacteriophages of *Ralstonia* species. Shotgun metagenomic sequencing was specifically selected over amplicon-based techniques because it provides unbiased coverage of both prokaryotic and viral communities, enabling simultaneous characterization of bacterial populations and their associated phage [33–35]. This approach allows for identification of functional genes related to plant-microbe interactions and potential antagonistic mechanisms against bacterial wilt pathogens that would remain undetected using targeted methods. This work provides baseline data that may inform future research on sustainable bacterial wilt management in Kenyan tomato production.

## 2 Materials and methods

### 2.1 Study design and sample collection

The study was conducted from August 2022 to October 2022 in the major tomato-growing counties of Kirinyaga, Kiambu, and Laikipia, located within the Mt. Kenya region in Kenya. Kirinyaga (0.6591° S, 37.3827° E), with elevations ranging from approximately 1100 meters in the lowlands to over 2000 meters on the slopes of Mt. Kenya, experiences a tropical wet and dry climate in the lower elevations and a subtropical highland climate in the higher areas. Average temperatures range from about 21°C in the lowlands to approximately 13–15°C at higher altitudes above 2000 meters. The region features primarily volcanic andisols on the mountain slopes and nitisols in the mid to lower elevations. Kiambu (1.1748° S, 36.8304° E), at approximately 1681.78 meters above sea level, experiences a subtropical highland climate with an average temperature of 20.82°C. The region consists of fertile volcanic soils, including andosols and nitisols, with a pH of approximately 6.3. Laikipia (0.3606° N, 36.7820° E), at approximately 1771.73 meters above sea level, experiences a climate ranging from semi-arid in the lower areas to subtropical highland in the higher elevations, with an average temperature of 17.21°C. The region features fertile black cotton soils (vertisols) in low-lying areas and sandy, well-drained red soils (ferralsols/nitisols) at higher altitudes.

A purposive sampling technique was used to collect soil samples from the rhizosphere of infected tomatoes at a depth of about 5–10 cm. The rhizosphere was specifically targeted because it represents the root-soil interface most directly influenced by plant-microbe interactions and is known to harbor microbial communities with antagonistic potential against soilborne pathogens including *R. solanacearum* [25–27]. Disease symptoms were assessed through visual observation following standard procedure as described by [36].Notably, the presence of wilted plants and/or dead plants without the presence of yellowing or spotting of the foliage and the appearance of a white, milky ooze streaming out of a freshly cut end of the discolored vascular tissue served as a confirmatory diagnostic test for this disease (Fig 1). The rhizosphere soils were also collected according to procedure described by [37], whereby tomato plants were uprooted, loose soil removed, and root-adhering soil collected with sterile tools. In total, 35 soil samples were collected from 23 sampling points (Fig 2; S1 Table). The soil samples were placed in sterile polyethylene bags separately to prevent drying out and labeled according to the sample points. All samples were stored in a cooler box with ice packs, maintaining a temperature of 4°C. Following collection, the samples were transported to the Institute for Biotechnology Research at Jomo Kenyatta University of Agriculture and Technology, Nairobi, Kenya, for further analysis. Additionally, the temperature of the soil at the sampling points was measured.

**Fig 1:**
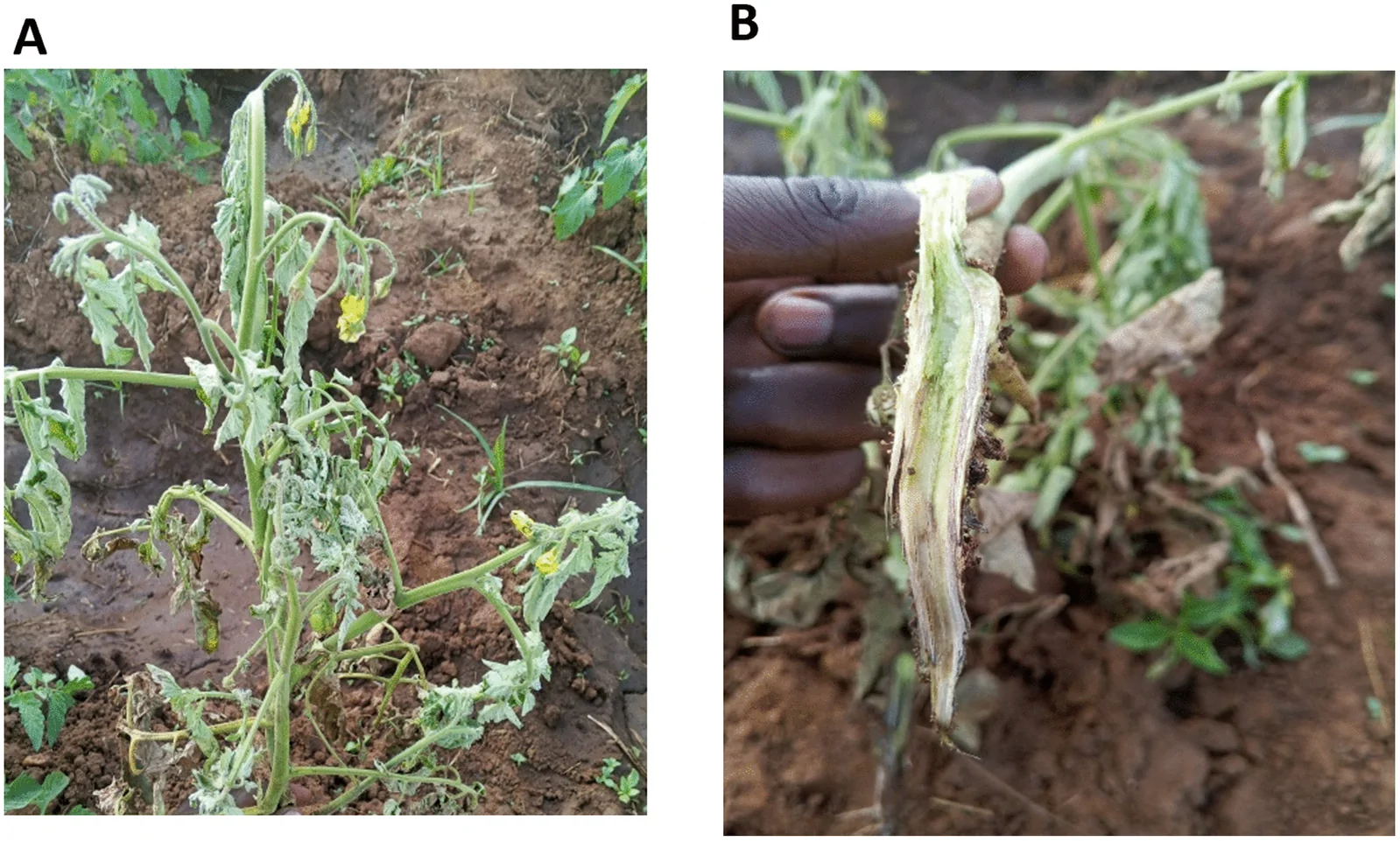
Symptoms of bacterial wilt in tomato at the sampling sites. (A) Wilted and collapsed tomato plants in the field. (B) Longitudinal section of a lower stem from a wilted plant showing discolouration of the vascular tissue near the base. GPS coordinates of all sampling points are given in S1 Table.

**Fig 2:**
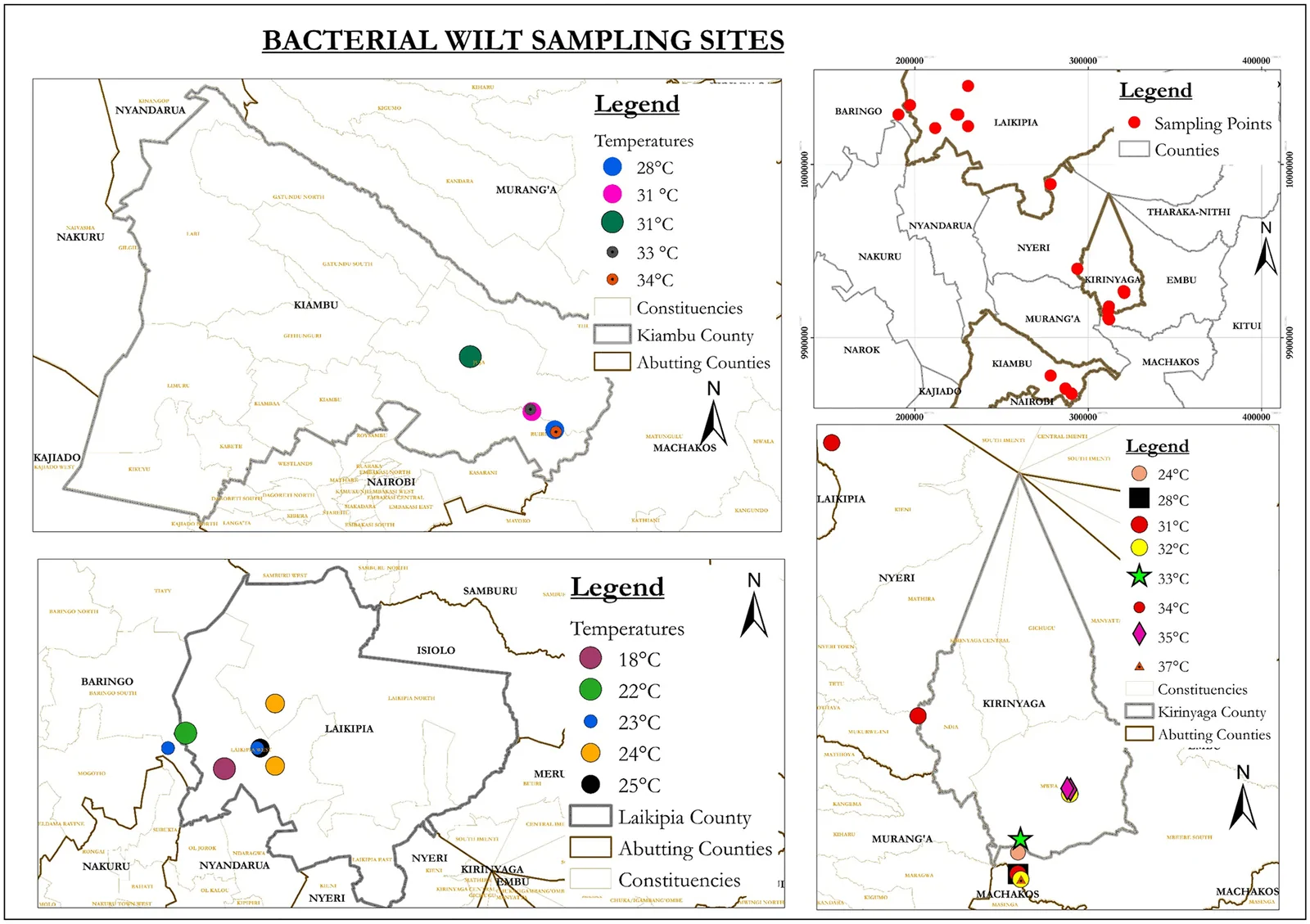
Geographic distribution of bacterial wilt sampling sites across Kenya. Maps show sampling locations in Kiambu County (top left), Laikipia County (bottom left), and Kirinyaga County (right panels), with sites color-coded by soil temperature. County and constituency boundaries are indicated. Administrative boundary shapefiles were obtained from the Kenya National Bureau of Statistics (KNBS). Map generated using QGIS version 3.44.

This study was conducted as part of a postgraduate research project at Jomo Kenyatta University of Agriculture and Technology (JKUAT). Field sampling was approved by the Institute for Biotechnology Research (IBR), JKUAT, which reviewed the research proposal, including the sampling protocol and farmer consent procedures, before the study began. Prior to sample collection, written informed consent was obtained from all farm owners at each sampling site using a standardised consent form. No other permits were required for this work, as the only approvals required for this institutional postgraduate project were the approval of IBR and the consent of the farm owners.

### 2.2 DNA extraction and sequencing approaches

Genomic DNA was extracted from soil samples using a modified phenol-chloroform protocol adapted from [38]. DNA integrity and quality were assessed through agarose gel electrophoresis to confirm suitability for downstream shotgun metagenomic sequencing. Following quality verification, extracted DNA from all samples was pooled to create a composite sample for sequencing. Pooling was performed to maximize metagenomic coverage across sampling sites and to obtain sufficient DNA concentration for library preparation. This approach captures the collective microbial diversity across the sampled agroecological zones.

Metagenomic shotgun libraries were prepared by random DNA fragmentation to 350 bp average insert size, followed by size selection, adapter ligation, and PCR amplification. Paired-end sequencing (2 × 150 bp) was performed on an Illumina NovaSeq at Molecular Research Laboratory and Pathology (MR DNA, USA), targeting a minimum of 20 million paired-end reads for adequate microbial community coverage. No virus-like particle enrichment or phage-specific DNA amplification was performed; viral sequences were identified computationally from the total metagenomic dataset.

### 2.3 Bioinformatics and data analysis

The metagenomic sequence data underwent quality control using FastQC v0.11.9 [39] and Trimmomatic v0.39 [40]. Assembly was done using MEGAHIT v1.2.9 [41]; and this assembly was used for DIAMOND/MEGAN. Because MEGAHIT contigs were short, reads were also assembled with metaSPAdes v3.15.5 [42]*, and the two contig sets were concatenated for Kraken2 classification and BLASTn confirmation.*

Taxonomic assignment was performed using DIAMOND [43] for protein-level alignment against the NCBI nr database, and then results were processed for taxonomic assignment using MEGAN6 [44]. Viral sequences were identified using Kraken2 v2.1.3 [45] against a viral reference database, and validated using BLASTn [46] against NCBI Viral RefSeq [47] (E-value <1 × 10^−5^).

DIAMOND and MEGAN assigned most *Ralstonia* sequences only to genus level. Species-level identification was therefore obtained by classifying all assembled contigs with Kraken2 [45] using the Standard-8 index, and contigs assigned to *Ralstonia* were confirmed by BLASTn [46] against a local database of 478,197 sequences, made up of an NCBI RefSeq bacterial subset supplemented with all 1,258 RefSeq Ralstonia assemblies (E-value ≤ 1 × 10^−5^; best hit by bitscore) A contig was considered species-confirmed when its best hit was the species assigned by Kraken2, with ≥97% identity and ≥50% query coverage; only contigs ≥200 bp were considered. Counts are numbers of contigs. Only BLASTn-confirmed species are reported in Fig 5; the Kraken2 output and confirmation status of each taxon are given in S5 Table.

**Fig 3:**
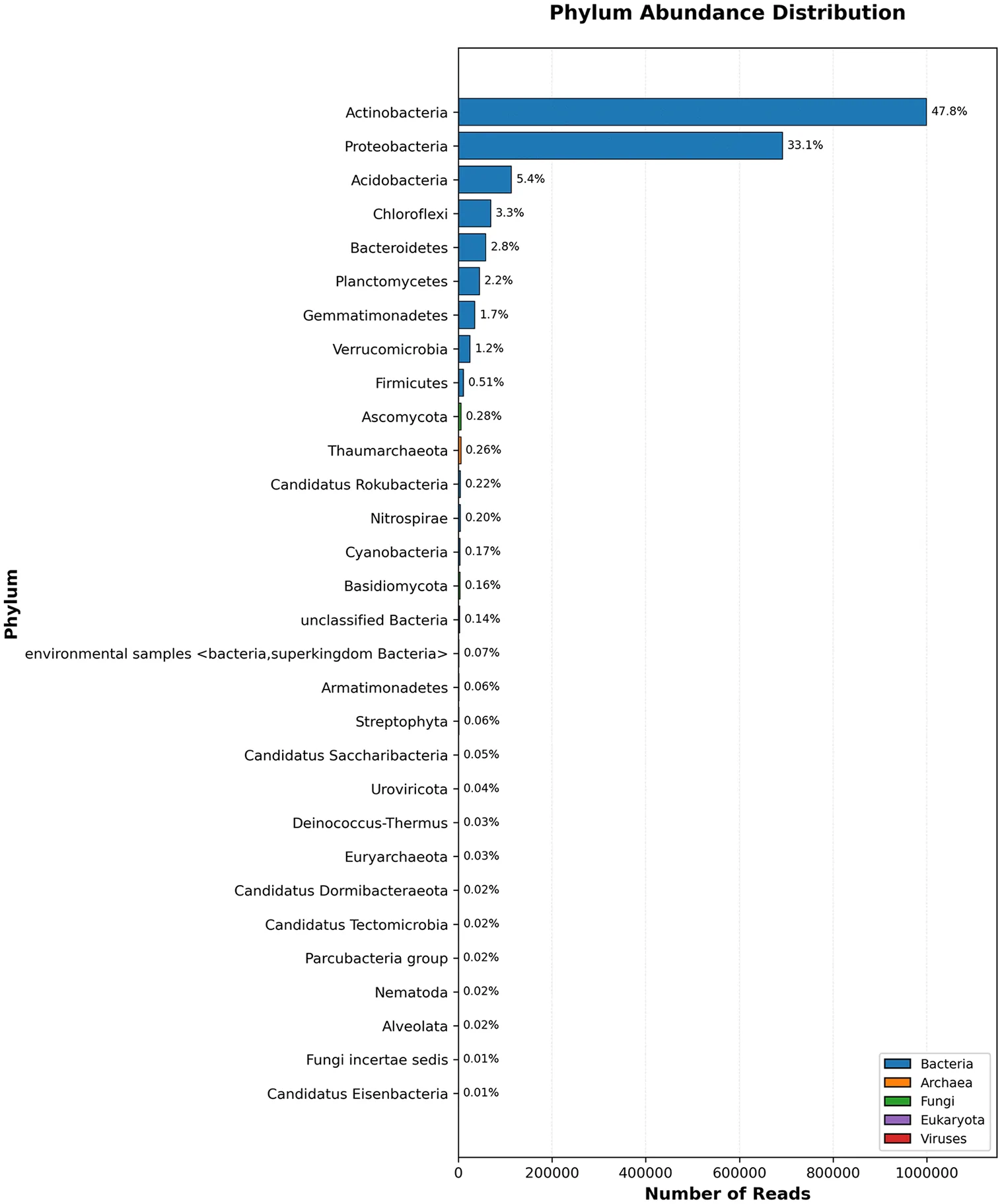
Phylum-level taxonomic composition of the soil microbial community. Horizontal bars represent the number of sequencing reads assigned to each phylum, with percentages indicating relative abundance. Phyla are color-coded by domain: Bacteria (blue), Archaea (orange), Fungi (green), Eukaryota (purple), and Viruses (red).

**Fig 4:**
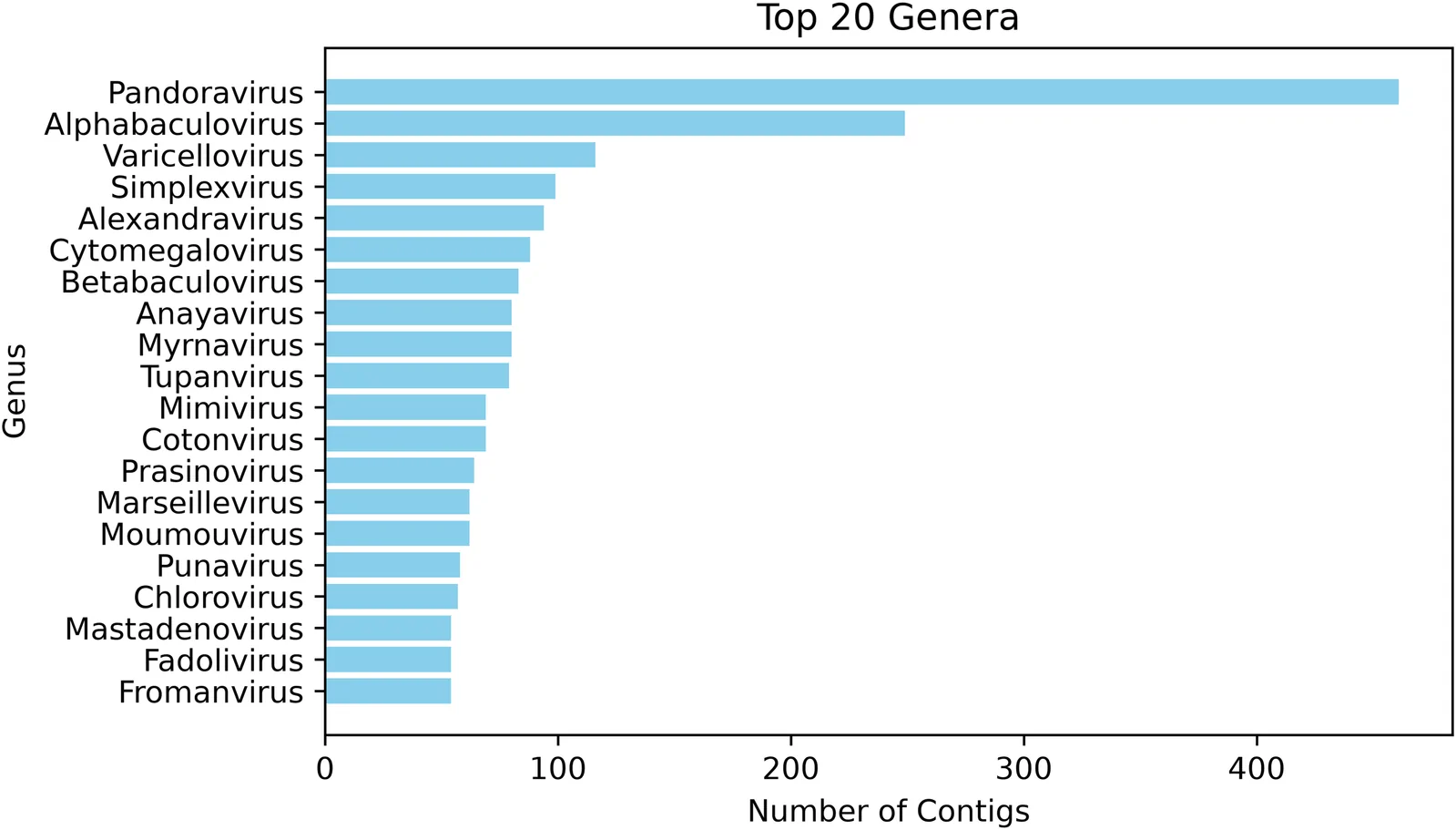
Distribution of the top 20 most abundant viral genera identified in the soil metagenome. Horizontal bars represent the number of contigs assigned to each viral genus by Kraken2.

Functional annotation utilized SEED [48], eggNOG [49], and InterPro [50] databases. Data visualization was performed using Matplotlib v3.x and NumPy v1.x in Python 3.x [51,52]. All bioinformatics scripts used in this analysis are publicly available on Zenodo (https://doi.org/10.5281/zenodo.23780945) and GitHub (https://github.com/Derleen123/Tomato-rhizosphere-metagenomics).

As DNA from all sampling sites was pooled into a single composite sample prior to sequencing, community diversity metrics requiring replicate samples (such as alpha and beta diversity indices) were not applicable. Microbial community composition was therefore assessed through relative abundance profiling of taxonomic classifications.

## 3 Results

### 3.1 Phylum-level taxonomic profile of soil microbiome

Taxonomic analysis at the phylum level identified 30 distinct phyla within the soil sample, representing a total of 2,089,233 reads. The bacterial community was dominated by Actinobacteria (47.8%) and Proteobacteria (33.1%), which together accounted for 80.96% of all sequences. Other notable bacterial phyla included Acidobacteria (5.4%), Chloroflexi (3.3%), Bacteroidetes (2.8%), Planctomycetes (2.2%), Gemmatimonadetes (1.7%), and Verrucomicrobia (1.2%). Archaeal sequences were represented by Thaumarchaeota (0.26%) and Euryarchaeota (0.03%). Low-abundance taxa included fungal phyla (Ascomycota, Basidiomycota and Fungi incertae sedis, 0.46% combined), metazoan sequences (Nematoda, 0.02%), and viral sequences (Uroviricota, 0.04%), contributing to the overall microbial diversity of the soil ecosystem (Fig 3; S3 Table). As a single pooled composite sample was analyzed, statistical comparisons between sampling sites were not performed. Community composition is therefore reported as relative abundance of classified sequences within the total metagenomic dataset.

### 3.2 Taxonomic profiling of viral sequences in the soil sample

Kraken2 assigned 11,715 contigs to 1,812 viral genera, of which the 20 most abundant accounted for 2,032 contigs (17.3%). Pandoravirus was the most abundant (461 contigs), followed by Alphabaculovirus (249), Varicellovirus (116), Simplexvirus (99) and Alexandravirus (94). The remaining genera ranged from 88 contigs (Cytomegalovirus) to 54 (Mastadenovirus, Fadolivirus and Fromanvirus). Five of the 20 are phage genera (Alexandravirus, Anayavirus, Myrnavirus, Punavirus and Fromanvirus); the rest are giant virus, baculovirus, herpesvirus and other eukaryotic virus genera (Fig 4; S4 Table).

### 3.3 Taxonomic profile of Ralstonia species in soil samples

Kraken2 assigned 269 contigs to *Ralstonia* (S5 Table) Of these, 172 had a Ralstonia best BLASTn hit and 64 were confirmed at species level: *R. insidiosa* (35), *R. pickettii* (26), *R. mannitolilytica* (2) and *R. wenshanensis* (1) (Fig 5; S5 Table). Contigs assigned to the *R. solanacearum* species complex were detected, but none met the species-level criteria. Most *Ralstonia* contigs were short (median 259 bp), which limits species-level resolution (S5 Table).

### 3.4 Functional classification

The functional profile of the microbial communities from the soil samples was assessed by integrating annotations from SEED, eggNOG, and InterPro2GO databases (Fig 6; S2 Table). The SEED database annotations showed a predominant representation of metabolic functions, energy production, protein processing, DNA processing, membrane transport and cellular processes. This indicates active nutrient cycling and microbial adaptation to environmental pressures. Stress response mechanisms and virulence-related functions were also identified, though at lower abundances, indicating the microbial community’s capacity to withstand environmental challenges. The InterPro2GO database revealed significant contributions from Molecular function and biological process categories, with cellular components also well represented. A high proportion remained unclassified, indicating untapped functional potential within this community. Functional annotation using eggNOG revealed metabolism as the dominant category, including pathways related to cellular processes, signaling, and information storage, indicating a well-adapted system for microbial growth and survival.

**Fig 5:**
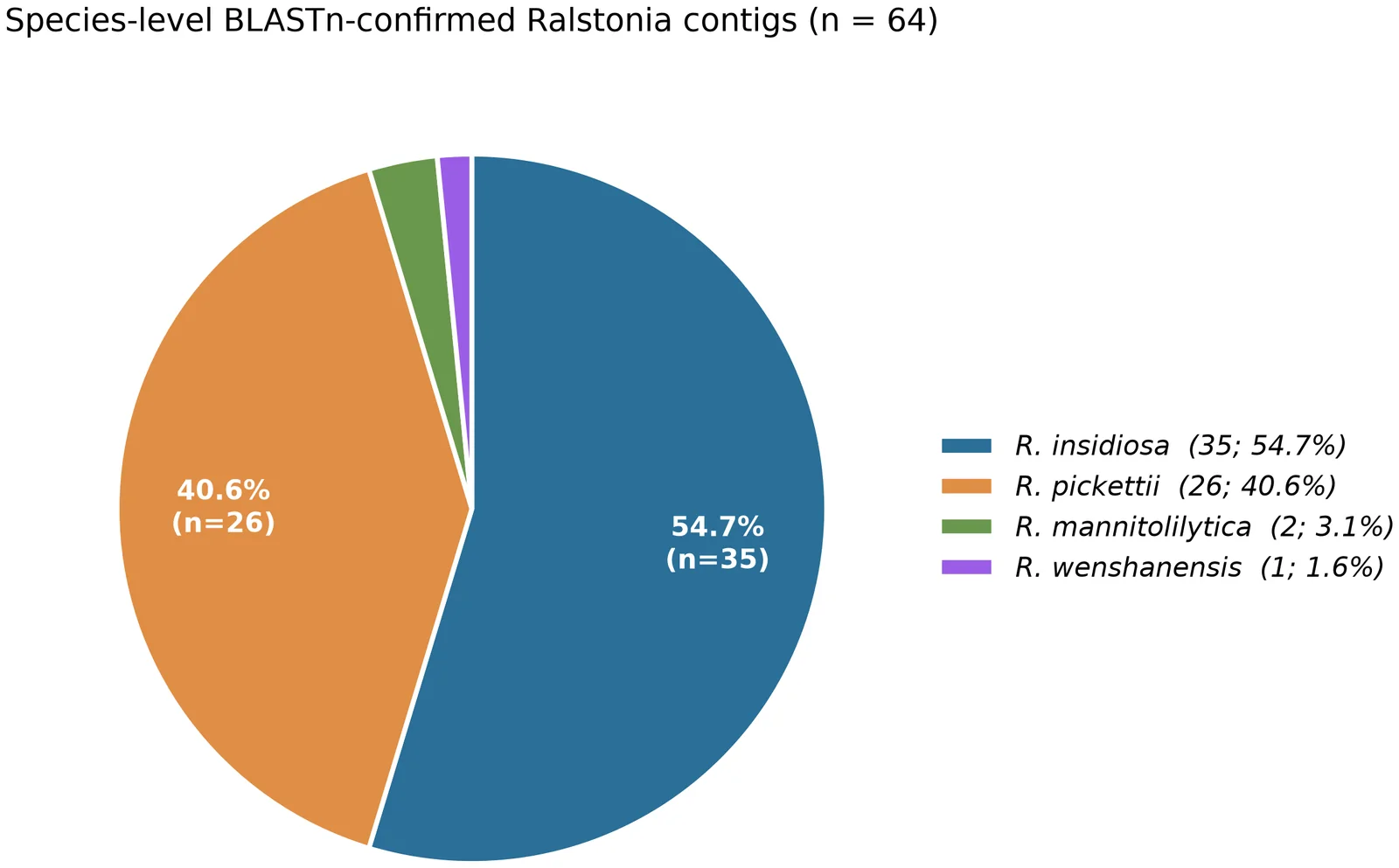
Species-level composition of BLASTn-confirmed *Ralstonia* contigs in the soil sample. Pie chart of *Ralstonia* contigs assigned to species by Kraken2 and confirmed by BLASTn (≥97% identity, ≥50% query coverage, contigs ≥200 bp; n = 64). Percentages are shares of species-confirmed contigs only, not overall abundance (S5 Table).

**Fig 6:**
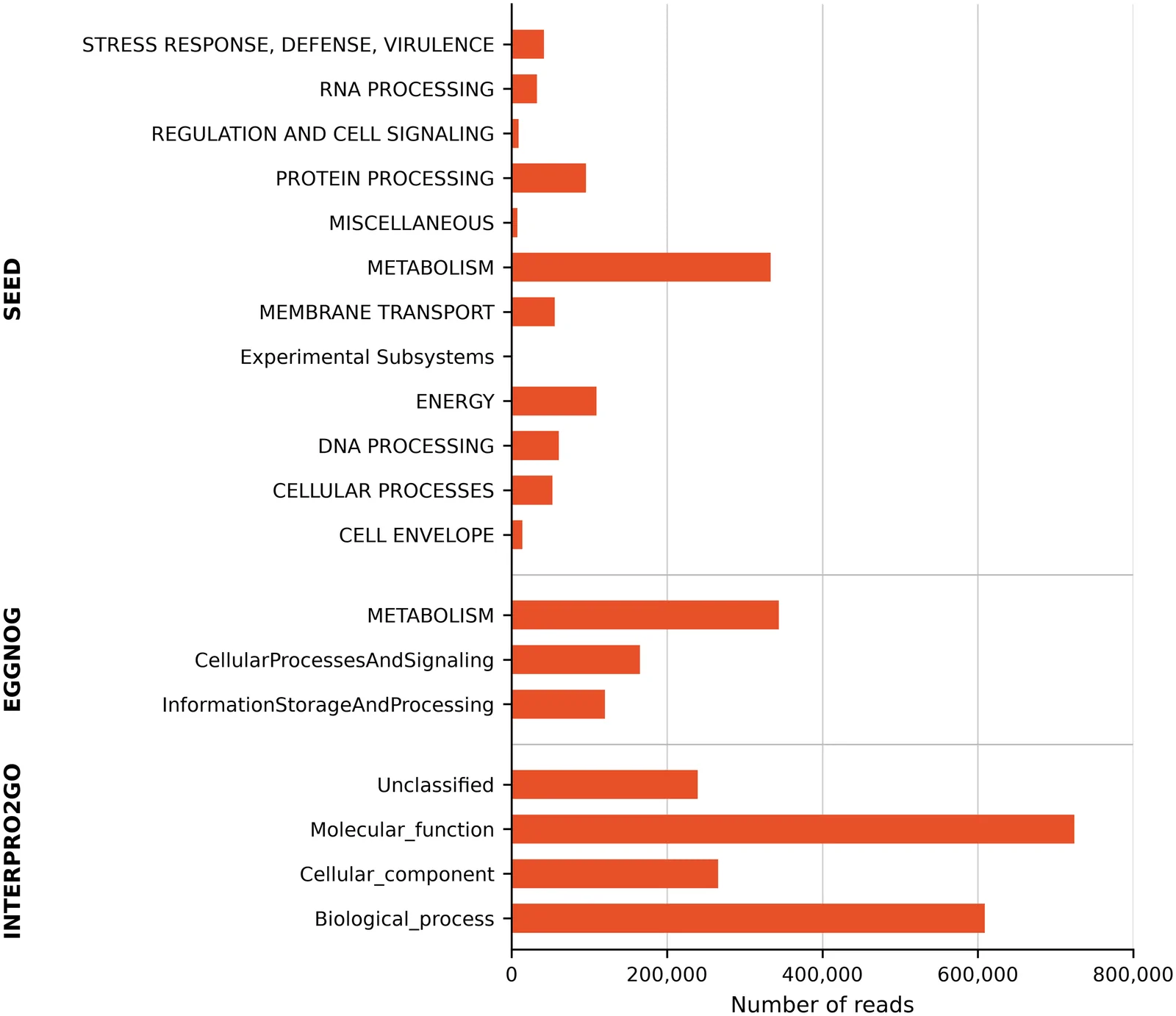
Functional annotation of tomato rhizosphere metagenomes. Horizontal bars indicate the number of sequencing reads assigned to each functional category.

## 4 Discussion

The metagenomic analysis of tomato rhizosphere microbiomes from major production regions in Kenya provides valuable insights into the microbial communities associated with bacterial wilt-infected tomato plants. Our findings reveal a high taxonomic diversity and functional potential that could inform sustainable management strategies for *Ralstonia solanacearum*.

The predominance of Actinobacteria and Proteobacteria is consistent with earlier research on agricultural soil microbiomes, which emphasizes the contributions of these phyla in the soil ecosystem [53–55]. Actinobacteria are also known for their antagonistic properties against soil-borne pathogens through the production of bioactive metabolites [56], while members of Proteobacteria participate in key ecological functions including nitrogen fixation and mineral cycling [57,58]. The high abundance of these phyla suggests potential natural suppressive mechanisms against pathogens, which could be harnessed for biocontrol applications [59,60]. The presence of Acidobacteria as the third most abundant phylum is notable, as these organisms are associated with organic matter decomposition and nutrient cycling, potentially influencing soil health and disease development [61,62].

Viral community characterization presented significant taxonomic challenges that required methodological optimization. DIAMOND protein homology searches coupled with MEGAN provided limited viral taxonomic resolution, consistent with known limitations of alignment-based approaches for divergent soil viromes. These limitations include extreme sequence divergence from reference databases, short gene lengths that reduce alignment confidence, and the prevalence of genes with no detectable homology [63–65]. Our DIAMOND+MEGAN approach yielded predominantly broad classifications, with 193 sequences assigned to “unclassified Siphoviridae” and minimal genus-level resolution. Furthermore, bacterial taxa (Streptomyces, Achromobacter, Microbacterium, and Firmicutes) were identified as viral classifications, likely reflecting prophage sequences or contamination in reference databases [66–70].

To address these limitations, we implemented Kraken2, a k-mer-based classifier utilizing exact nucleotide matches rather than protein alignment, which provides superior performance for divergent and novel soil viral sequences [45,64,71]. This approach yielded improved taxonomic resolution for the soil virome. From 3,353,930 assembled contigs, Kraken2 identified 13,741 viral classifications, of which 959 (6.98%) achieved BLAST validation (E-value < 1 × 10^−5^), representing 375 distinct viral families, genera, and species. While this confirmation rate may appear modest, it aligns with expectations for soil viral communities, where most sequences represent novel or highly divergent lineages with limited database representation [72,73]. The viral community exhibited considerable taxonomic diversity, with Pandoravirus, Alphabaculovirus, and Varicellovirus as the most abundant genera. The predominance of tailed bacteriophages (order Caudoviricetes) reflects typical soil virome composition, where these morphotypes dominate owing to their broad bacterial host ranges and environmental stability [74–76].

Of particular significance to this study’s focus on bacterial wilt management, our analysis detected sequences matching four Ralstonia phage genomes through BLAST validation: Ralstonia phage RSL1, Ralstonia phage RSIBR3, Ralstonia phage Anchaing, and Ralstonia phage Cimandef (S6 Table). The detection of RSL1 is especially noteworthy, as this phage has been previously characterized for its lytic activity against *R. solanacearum* and proposed as a biocontrol candidate [77]. While our metagenomic analysis confirms the presence of these phage sequences in Kenyan agricultural soils, it does not directly demonstrate lytic activity or antagonistic interactions with local Ralstonia strains. The biocontrol potential of these phages requires validation through laboratory isolation and host-range assays. Phages isolated from these soils might be well suited to local conditions [78,79] but this remains to be tested.

The high proportion of unclassified and novel viral sequences in our dataset underscores the vast unexplored viral diversity in agricultural soils and highlights the need for expanded viral reference databases specifically targeting soil ecosystems [73]. The functional characterization of these novel phages, particularly their host ranges, infection dynamics, and environmental persistence, represents a critical direction for future research aimed at developing phage-based biocontrol strategies for bacterial wilt management. BLASTn confirmed four Ralstonia species within the rhizosphere samples, with *R. insidiosa* being the most frequent (35 of 64 species-confirmed contigs). While *R. insidiosa* is not a known plant pathogen like *R. solanacearum*, it has been implicated in opportunistic infections in humans, particularly in hospital settings [80]. *R. insidiosa and R. pickettii are also common in water systems, so contamination cannot be excluded in the absence of a negative control* [81,82]. *Contigs assigned to, members of the Ralstonia solanacearum species complex (RSSC; R. solanacearum, R. pseudosolanacearum, R. syzygii), the well-established plant pathogens responsible for bacterial wilt* [14,83–87], were also detected. This is consistent with the symptomatic plants sampled and with previous reports of high incidence in tomato-growing regions [12]. However, these contigs were too short (median 259 bp) for species-level confirmation, so these data neither confirm nor exclude wilt-causing strains. The coexistence of multiple Ralstonia species highlights the complexity of bacterial wilt management, emphasizing the need for integrated strategies that account for a diverse and potentially interacting pathogen community. The detection of sequences matching *Ralstonia* phages RSL1, RSIBR3, Anchaing, and Cimandef alongside *Ralstonia* species in the same dataset suggests a possible ecological interaction that may influence pathogen populations, and warrants further study as a basis for phage-based biocontrol development.

The functional annotation of the metagenome revealed predominant metabolic pathways, energy production mechanisms, and stress response systems, indicating a highly adaptive microbial community [88]. The presence of genes related to virulence factors and stress responses suggests potential involvement in pathogen-host interactions and environmental adaptation. Similar functional profiles have been reported in suppressive soils where natural disease control mechanisms operate [89,90]. The abundance of metabolism-related genes, particularly those involved in carbon and nitrogen cycling, indicates active nutrient turnover processes that could influence plant health and pathogen suppression [91–94].

The high soil temperatures recorded at sampling sites may contribute to the proliferation of Ralstonia species, as these pathogens typically thrive under warm conditions [12,20,95]. Climate change-induced temperature increases may further exacerbate bacterial wilt incidence in these regions, necessitating adaptive management strategies [18]. The detection of multiple stress response genes in our functional analysis suggests that the rhizosphere microbiome has developed mechanisms to cope with these challenging environmental conditions [96,97].

The comprehensive metagenomic approach employed in this study provides an understanding of the rhizosphere microbiome that would not be possible with culture-dependent methods or targeted amplicon sequencing. By simultaneously characterizing the bacterial, fungal, and viral components of the soil community, we gain insights into the complex ecological interactions that may influence disease development and suppression. The detection of *Ralstonia* phage sequences suggests that these soils could be a source of phages for future biocontrol studies, which might support locally adapted strategies that reduce reliance on chemical control agents and enhance the sustainability of tomato production in Kenya.

While our metagenomic approach provides a comprehensive view of taxonomic and functional diversity, it does not directly demonstrate the activity of identified microorganisms or their interactions with tomato plants under field conditions. Future research should incorporate transcriptomic and metabolomic approaches to assess the expression of identified functional genes and their products in the context of bacterial wilt progression and suppression. Comparative metagenomic analysis of healthy versus bacterial wilt-infected rhizosphere microbiomes would also be valuable in identifying pathogen-associated microbial shifts and potential disease-suppressive community members that were not distinguishable in this single-condition study. Additionally, isolation and characterization of the identified phages, particularly RSL1, RSIBR3, Anchaing, and Cimandef, would be essential to validate their lytic activity against local Ralstonia strains and evaluate their potential as biocontrol agents under Kenyan agricultural conditions. Host range determination, virion stability under field conditions, and compatibility with existing agricultural practices should be prioritized in future phage biocontrol development efforts.

This study provides valuable baseline data on the composition and functional potential of tomato rhizosphere microbiomes in Kenya’s major production regions. The detection of sequences matching four known *Ralstonia* phages, along with diverse bacteria with potential antagonistic activity, identifies candidates for future testing as locally adapted biological control strategies against bacterial wilt. These findings contribute to our understanding of the complex ecological relationships in the rhizosphere and may inform sustainable management practices that enhance tomato production while reducing environmental impacts associated with conventional control methods.

## 5 Conclusion

This study provides an extensive metagenomic characterization of the tomato rhizosphere microbiome in key growing areas of Kenya and uncovered diverse microbial and viral communities with possible relevance for disease control. Several *Ralstonia* species were identified, along with sequences matching *Ralstonia* phages RSL1, RSIBR3, Anchaing, and Cimandef. These findings suggest a possible phage-pathogen relationship in the rhizosphere and provide a starting point for isolating phages and testing their potential for biocontrol of bacterial wilt. The dominance of Actinobacteria and Proteobacteria, which include many beneficial taxa and the presence of genes involved in nutrient cycling and stress responses indicate a functionally rich microbiome that could support plant health and resilience. These findings are valuable for researchers aiming to design biological control strategies guided by rhizosphere microbiome composition, for farmers seeking sustainable alternatives to chemical control, and for policymakers promoting environmentally sound agricultural practices. Further work on rhizosphere microbiomes and *Ralstonia* phages may inform more resilient and sustainable tomato production systems in Kenya and similar agroecological contexts.

## Supporting information

S1 Table GPS coordinates and soil temperature of the 23 tomato rhizosphere sampling points. (XLSX)

S2 Table Functional annotation of the tomato rhizosphere metagenome (data underlying Fig 6). (XLSX)

S3 Table Phylum-level taxonomic composition of the tomato rhizosphere metagenome (data underlying Fig 3). (XLSX)

S4 Table Top 20 most abundant viral genera in the tomato rhizosphere metagenome (data underlying Fig 4). (XLSX)

S5 Table Kraken2 classification of Ralstonia in the tomato rhizosphere metagenome and BLASTn confirmation status (BLASTn-confirmed counts underlie Fig 5). (XLSX)

S6 Table Viral reference genomes matched by BLASTn-validated Kraken2 contigs in the tomato rhizosphere metagenome (Ralstonia phages highlighted). (XLSX)
